# EEG Characteristics of Dementia With Lewy Bodies, Alzheimer’s Disease and Mixed Pathology

**DOI:** 10.3389/fnagi.2018.00190

**Published:** 2018-07-03

**Authors:** Jessica J. van der Zande, Alida A. Gouw, Inger van Steenoven, Philip Scheltens, Cornelis Jan Stam, Afina W. Lemstra

**Affiliations:** ^1^VU Medical Center Alzheimer Center, Amsterdam, Netherlands; ^2^Department of Clinical Neurophysiology, VU Medical Center, Amsterdam, Netherlands

**Keywords:** dementia with Lewy bodies (DLB), Alzheimer’s disease (AD), EEG, spectral analysis, differential diagnosis

## Abstract

**Introduction**: Previous studies on electroencephalography (EEG) to discriminate between dementia with Lewy bodies (DLB) and Alzheimer’s disease (AD) have been promising. These studies did not consider the pathological overlap of the two diseases. DLB-patients with concomitant AD pathology (DLB/AD+) have a more severe disease manifestation. The EEG may also be influenced by a synergistic effect of the two pathologies. We aimed to compare EEG characteristics between DLB/AD+, “pure” DLB (DLB/AD−) and AD.

**Methods**: We selected probable DLB patients who had an EEG and cerebrospinal fluid (CSF) available, from the Amsterdam Dementia Cohort (ADC). Concomitant AD-pathology was defined as a CSF tau/Aβ-42 ratio > 0.52. Forty-one DLB/AD+ cases were matched for age (mean 70 (range 53–85)) and sex (85% male) 1:1 to DLB/AD− and AD-patients. EEGs were assessed visually, with Fast Fourier Transform (FFT), network- and connectivity measures.

**Results**: EEG visual severity score (range 1–5) did not differ between DLB/AD− and DLB/AD+ (2.7 in both groups) and was higher compared to AD (1.9, *p* < 0.01). Both DLB groups had a lower peak frequency (7.0 Hz and 6.9 Hz in DLB vs. 8.2 in AD, *p* < 0.05), more slow-wave activity and more prominent disruptions of connectivity and networks, compared to AD. No significant differences were found between DLB/AD+ and DLB/AD−.

**Discussion**: EEG abnormalities are more pronounced in DLB, regardless of AD co-pathology. This emphasizes the valuable role of EEG in discriminating between DLB and AD. It suggests that EEG slowing in DLB is influenced more by the α-synucleinopathy, or the associated cholinergic deficit, than by amyloid and tau pathology.

## Introduction

Dementia with Lewy Bodies (DLB) is the most common form of dementia in the aging population after Alzheimer’s disease (AD; Zaccai et al., 2005). DLB is characterized clinically by cognitive decline accompanied by visual hallucinations, parkinsonism, fluctuations of cognition and/or sleep disturbances (McKeith et al., 2017). Adequate diagnosis is important for optimal clinical management. Yet in clinical practice this can be challenging, and DLB tends to be underdiagnosed (Toledo et al., 2013). The most difficult discrimination is from AD, due to coinciding clinical features, as well as pathological overlap (Jellinger, 2004). The pathological substrate of DLB is aggregation of α-synuclein in Lewy bodies and neurites. However, up to 50%–80% of patients with DLB have co-existing Alzheimer-pathology, i.e., amyloid plaques and neurofibrillary tangles (Jellinger and Attems, 2008; Howlett et al., 2015).

The DLB patients with concomitant AD pathology (DLB/AD+) represent a specific diagnostic challenge since abnormal AD biomarkers (such as Aβ-42, tau and p-tau in cerebrospinal fluid (CSF), and amyloid-PET (Scheltens et al., 2016)) can lead to an incorrect diagnosis of AD (Jellinger, 2004). Decreased striatal dopamine transporter (DAT) binding on SPECT can be valuable (McCleery et al., 2015) as well as imaging of the postganglionic sympathetic cardiac innervation (^123^iodine-MIBG; Treglia and Cason, 2012). However, these methods are costly and not always available in a clinical setting. Furthermore, false-negative DAT SPECT scans early in the disease course of DLB have been described (van der Zande et al., 2016).

Electroencephalography (EEG) has been widely studied for the (early) diagnosis of DLB, and has been implemented in the recently revised diagnostic criteria for DLB as a supportive biomarker (Bonanni et al., 2008, 2016; Roks et al., 2008; Lee et al., 2015; Dauwan et al., 2016b; McKeith et al., 2017). It is a low cost, non-invasive and widely available diagnostic test that provides a functional measure of neuronal and synaptic integrity. In EEG-studies, DLB patients showed decreased reactivity of the background activity and pronounced slow-wave and paroxysmal activity, such as frontal intermittent rhythmic delta activity (FIRDA). These features can be detected by visual analysis (Roks et al., 2008; Liedorp et al., 2009; Lee et al., 2015). When EEG is analyzed quantitatively (qEEG), DLB has been associated with increased power in the theta and delta frequency bands, a low dominant frequency, high dominant frequency variability (Bonanni et al., 2008; Cromarty et al., 2016) and prominent disruptions of functional connectivity compared to AD and controls (van Dellen et al., 2015; Dauwan et al., 2016a). Additionally, automated analysis of combined (q)EEG features has shown good discriminative value (accuracy >85%) between DLB and AD (Bonanni et al., 2016; Dauwan et al., 2016b).

However, previous EEG-studies did not take into account concomitant AD pathology in DLB. Compared to “pure” DLB, DLB/AD+ patients have shown a more severe disease manifestation (Howlett et al., 2015; Lemstra et al., 2017) and a synergistic effect of α-synuclein and amyloid and tau pathology is suspected (Howlett et al., 2015). This could be reflected by more severe EEG abnormalities in this group. Alternatively, since in AD the EEG abnormalities do not seem as extensive as in DLB (Kai et al., 2005; Liedorp et al., 2009), in DLB/AD+ the EEG results could be “in between” AD and DLB. In other words, the concomitant AD-pathology could cause part of the more severe clinical symptoms, while the EEG does not reflect this. We hypothesized that concomitant AD-pathology in DLB influences EEG measures and aimed to compare EEG characteristics between three groups: pure DLB, DLB/AD+ and AD patients.

## Materials and Methods

### Study Population

Patients were selected from the Amsterdam Dementia Cohort (ADC) if they fulfilled the 2005 diagnostic criteria for probable DLB (McKeith et al., 2005) and had both EEG and a lumbar puncture performed. The ADC is a clinical cohort built from patients who visited our memory clinic for a one-day diagnostic screening including medical history, physical and neurological examination, blood and CSF analysis, EEG and structural brain imaging (van der Flier et al., 2014). Diagnoses were made in a multidisciplinary setting according to clinical consensus criteria (McKeith et al., 2005; McKhann et al., 2011). Additional ^123^I[FP-CIT]-SPECT imaging was performed at the discretion of the clinical team.

From patients who visited the memory clinic between May 2003 and September 2015, 121 DLB-patients met the criteria mentioned above. Concomitant AD-pathology (DLB/AD+) was defined as a CSF tau/Aβ-42 ratio >0.52, which has been associated with a stable diagnosis of AD in a large memory clinic cohort (Duits et al., 2014). Forty-one patients who fulfilled the clinical criteria for probable DLB, had a CSF tau/Aβ-42 ratio >0.52. These patients were defined as the mixed pathology (DLB/AD+) group. DLB/AD+-patients were matched for age and sex on a group level with 41 “pure” DLB patients and 41 AD-patients. In 28 cases, a ^123^I[FP-CIT]-SPECT was available. AD was diagnosed according to NINCDS-ADRDA criteria (McKhann et al., 2011), and in all AD-patients the CSF tau/Aβ-42 ratio was >0.52 (Duits et al., 2014).

Clinical data were collected prospectively at the initial visit. The mini mental state examination (MMSE; Cockrell and Folstein, 1988) score was used as a global cognitive measure. Hallucinations were scored according to the neuropsychiatric inventory (NPI; Cummings, 1997), extrapyramidal signs according to a preformatted checklist (based on the presence or absence of bradykinesia, rigidity and/or tremor).

### Ethics

The local medical ethical committee, METc VU University Medical Center, approved the study. All patients gave written informed consent for retrospective use of their clinical data.

### CSF Analysis

CSF was obtained via lumbar puncture. The procedure is described in more detail elsewhere (van der Flier et al., 2014). In short, CSF was collected in polypropylene tubes, centrifuged at 1800 *g* for 10 min at 4°C, processed and stored in aliquots of 0.5 ml at 80°C. Amyloid-β42, total tau and p-tau were measured by INNOTEST Double sandwich ELISAs.

### EEG Recording

All subjects underwent a 20-min resting-state EEG with O.S.G. digital equipment (Brainlab or BrainRT; O.S.G. B.V. Belgium). Twenty-one scalp electrodes were placed according to the international 10-20 system on the following locations: Fp2, Fp1, F8, F7, F4, F3, A2, A1, T4, T3, C4, C3, T6, T5, P4, P3, O2, O1, Fz, Cz and Pz. Electrode impedance was <5 kOhm. The EEG was filtered with a time constant of 1 s and a low pass filter of 70 Hz online. The sample frequency was 500 Hz. Recording took place in a slightly reclined chair. When necessary, EEG technicians used sound stimuli to keep the patients awake. Source derivation was used as a reference (Hjorth, 1975), and the data was band-pass filtered in six frequency bands: delta (0.5–4 Hz) theta (4–8 Hz), alpha-1 (8–10 Hz), alpha-2 (10–13 Hz), beta (13–30 Hz) and gamma (30–48 Hz). Oscillations <0.5 Hz and >30 Hz were excluded from further analyses because of the expected artifacts from muscle and eye movement (Hagemann and Naumann, 2001; Whitham et al., 2007). Four artifact-free epochs of approximately 10 s per patient, sufficient for quantitative analyses (Gasser et al., 1985; van Diessen et al., 2015) recorded in an awake resting-state with eyes closed, were visually selected (according to a standard operating procedure).

### Visual EEG Assessment

**Figure 1:**
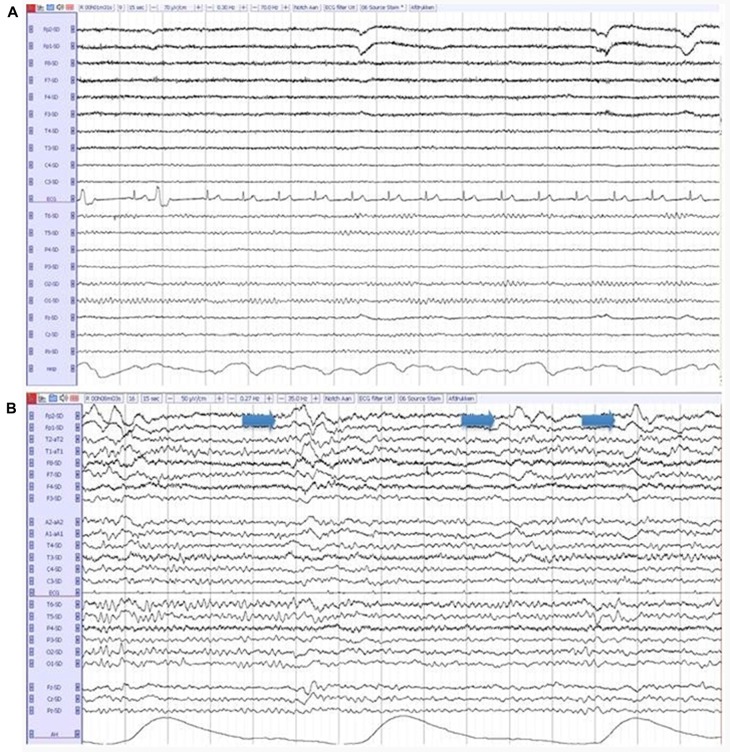
**(A)** A normal electroencephalography (EEG) in a patient with Alzheimer’s disease (AD). **(B)** An abnormal EEG in a patient with dementia with lewy bodies (DLB), showing diffuse slow-wave activity and frontal intermittent rhythmic delta activity (FIRDA; arrows).

The entire 20-min EEG registrations were visually assessed by certified clinical neurophysiologists, without knowledge of clinical information except for age, sex and medication use, according to a standardized visual rating scheme, which includes the severity of EEG abnormalities (on a 5-point scale: 1 = normal EEG, 2 = mildly abnormal, 3 = moderately abnormal, 4 = severely abnormal, 5 = iso-electric) and the presence of focal, diffuse and epileptiform abnormalities. Focal abnormalities were defined as sharp or slow waves, present in one or more EEG leads. Diffuse abnormalities consisted of a posterior dominant frequency below 8 Hz, diffuse slow wave activity or decreased reactivity of the background pattern to eye opening. Epileptiform discharges were defined as spikes, spike-and-slow-wave-complexes or sharp-and-slow-wave-complexes present in one or more EEG leads (Liedorp et al., 2009). The presence of FIRDA, Figure 1, was analyzed as a separate variable, because of the relatively high occurrence in DLB described in literature (Roks et al., 2008; Lee et al., 2015).

### Quantitative and Automated EEG Analysis

EEG data were converted to American Standard Code for Information Interchange format and loaded into BrainWave software, version 0.9.152.2.7^1^ to perform quantitative analyses. Fast Fourier transformation (FFT) was used to calculate relative power per frequency band and peak frequency per electrode. To indicate the ratio between slow-wave and fast-wave activity, we calculated the theta/alpha ratio: theta/(theta + alpha1 + alpha2).

MATLAB 2011a (MathWorks Inc., Natick, MA, USA) was used to configure head plots showing relative power per frequency band per channel. To assess the variability of the global peak frequency, the four selected epochs (of 8–10 s EEG signal) were divided into pieces of 2 s, resulting in 16 epochs per patient. The standard deviation of the peak frequency per patient was calculated as a measure of variability.

The strength of functional connectivity was assessed with the Phase Lag Index (PLI). This measure of functional connectivity, previously described in more detail (Stam et al., 2007), ranges between 0 (no phase locking) and 1 (complete synchronization) and was calculated for all electrode pairs per subject. Based on previous literature describing differences between DLB and AD, PLI was calculated for the alpha frequency band (8–13 Hz; van Dellen et al., 2015). A minimum spanning tree (MST) network was generated from the weighted adjacency matrix of PLI values and topology measures were calculated: highest degree (measure of regional importance, regions with a high degree may be considered “hubs”), leaf number (measure of network organization, describes to what extent a network is dependent on hubs), diameter (measure of the efficiency of network organization) and tree hierarchy (measure of hierarchy in network organization; van Dellen et al., 2015).

Finally, all qEEG data from all patients were entered as different variables or “features” in a random forest machine-learning algorithm using BrainWave. This method is described in detail elsewhere (Dauwan et al., 2016b). In short random forest is a classification method based on decision trees. Each decision tree in the random forest is built using a bootstrap sample (i.e., new training set), with replacement, consisting of 2/3 of the original data, and is tested on the remaining 1/3 of the data. Consequently, in random forest the cross-validation is done internally and there is no need for a separate test set to estimate the generalization error of the training set (Breiman, 1999). Furthermore each new training set of features is randomly drawn from the original dataset of features. This bootstrap aggregating (i.e., bagging), and random feature selection help in reducing the variance of the model, avoid overfitting, and result in uncorrelated trees. An advantage of ensembled decision tree methods is that feature selection is also done internally, that is the algorithm can identify from a large set of input the features that are really useful for the classification (Geurts et al., 2009). It is therefore possible to score the importance of a feature to the classification by means of the variable importance (VIMP) score. All our 25 available qEEG features were entered in this order (listed in Table 1): Delta power: lowest-mean-highest of the 21 channels; theta power: lowest-mean-highest; alpha1 power: lowest-mean-highest; alpha2-power: lowest-mean-highest; beta power: lowest-mean-highest; peak frequency: lowest-mean-highest, theta/alpha ratio; PLI (alpha band): lowest-mean-highest, MST (alpha band). Three automated classifiers are developed to divide patients between two diagnostic groups (DLB/AD+ vs. AD, DLB/AD− vs. AD and DLB/AD+ vs. DLB/AD−). The accuracy, sensitivity and specificity of the classifier were calculated, as well as a VIMP score per feature, which reflects the relative contribution of that feature to the classifier. A high VIMP score means a given EEG feature is important for the discrimination between the two diagnoses (Dauwan et al., 2016b).

**Table 1:** Features included in the random forest algorithm.

1	Lowest delta power	14	Mean beta power
2	Mean delta power	15	Highest beta power
3	Highest delta power	16	Lowest peak frequency
4	Lowest theta power	17	Mean peak frequency
5	Mean theta power	18	Highest peak frequency
6	Highest theta power	19	Theta/alpha ratio
7	Lowest alpha-1 power	20	Lowest PLI (alpha band)
8	Mean alpha-1 power	21	Mean PLI (alpha band)
9	Highest alpha-1 power	22	Highest PLI (alpha band)
10	Lowest alpha-2 power	23	Highest degree
11	Mean alpha-2 power	24	Leaf number
12	Highest alpha-2 power	25	Tree hierarchy
13	Lowest beta power		

### Statistical Analysis

Statistical analysis was carried out using SPSS (IBM, version 22). The groups were matched using SPSS case-control matching. Group differences in demographical, clinical data and EEG outcome measures were assessed using ANOVA (with Bonferroni corrected *post hoc* tests) and, in the case of non-normally distributed data, Kruskal Wallis tests. Linear regression was used to determine possible confounders or effect modifiers (age, sex, symptom duration, MMSE score and medication use) and when necessary, results were corrected with these variables added as covariates. A probability value of <0.05 was considered statistically significant.

## Results

### Patient Characteristics

Demographic and clinical characteristics are summarized in Table 2.

**Table 2:** Patient characteristics.

	DLB/ADC+	DLB/AD−	AD
	(*n* = 41)	(*n* = 41)	(*n* = 41)
Age, years	71 (6.6)	69 (6.3)	69 (7.1)
Symptom duration, years	2.6 (1.8)	3.6 (2.6)	3.4 (2.3)
Sex, male (%)	31 (76)	37 (90)	36 (88)
Hallucinations (%)	18 (44)	11 (27)	2 (7)^ab^
Extrapyramidal signs (%)	17 (50)	30 (77)^c^	1 (3)^ab^
	*n* = 34	*n* = 39	*n* = 37
*Medication*	12 (30)	9 (22)	7 (17)
*cholinesterase inhibitor*	3 (7)	3 (7)	6 (14)
*benzodiazepine*	8 (20)	3 (7)	1 (2)^a^
*antidepressant*	5 (12)	1 (2)	0
*antipsychotic*	2 (5)	2 (5)	0
MMSE	20 (5.6)	24 (3.6)^c^	22 (5.1)
	*n* = 40		*n* = 39
CSF AB42 pg/ml	501 (136)	811 (263)^c^	461 (134)^b^
median (IQR)	479 (215)	774 (385)	461 (159)
CSF total TAU, pg/ml	540 (260)	242 (79)^c^	640 (335)^b^
median (IQR)	496 (319)	245 (94)	570 (262)
CSF pTAU, pg/ml	73 (32)	41 (13)^c^	91 (39)^b^
median (IQR)	65 (35)	42 (13)	82 (43)
^123^I[FP-CIT]-scan available	11 (27%)	17 (41%)	

*Data are mean (SD) or n(%) unless otherwise specified. MMSE = mini mental state examination, CDR = clinical dementia rate, CSF = cerebrospinal fluid. ^a^p < 0.05 DLB/AD+ vs. AD, ^b^*p* < 0.05 DLB/AD− vs. AD, ^c^p < 0.05 DLB/AD+ vs. DLB/AD−*.

In DLB/AD− and DLB/AD+ more hallucinations and extrapyramidal signs were present compared to AD. DLB/AD+-patients had a lower MMSE score and used more antidepressants and benzodiazepines, compared to AD. Twenty-eight DLB-patients underwent DAT-SPECT imaging, which was abnormal in all cases.

### Visual EEG

Visual EEG scores are shown in Table 3.

**Table 3:** Visual electroencephalography (EEG) characteristics.

	DLB/AD+	DLB/AD−	AD
	(*n* = 41)	(*n* = 41)	(*n* = 41)
Severity score, mean (*SD*)	2.7 (0.5)	2.7 (0.5)	1.9 (0.8)^ab^
Focal abnormalities *n* (%)	32 (78)	28 (68)	22 (53)^a^
Diffuse abnormalities *n* (%)	36 (88)	38 (93)	16 (39)^ab^
Epileptiform abnormalities *n* (%)	1 (2)	0	1 (2.4)
FIRDA *n* (%)	8 (20)	12 (29)	1 (2)^ab^

*Data are mean (SD) or n(%), FIRDA, frontal intermittent rhythmic delta activity. ^\textsl a^*p* < 0.05 DLB/AD+ vs. AD, ^\textsl b^*p* < 0.05 DLB/AD− vs. AD (ANOVA)*.

The EEG severity score was significantly lower in both DLB-groups compared to AD (2.7 vs. 1.9, *p* < 0.001). Both the DLB/AD+ and the “pure” DLB group differed from the AD patients in the presence of diffuse abnormalities (present in 88% of DLB/AD+, 93% of DLB/AD−, 39% of AD, *p* < 0.001) and FIRDA (present in 20% of DLB/AD+, 29% of DLB/AD−, 2% of AD, *p* = 0.03).

#### Quantitative EEG—Spectral Analysis

**Figure 2:**
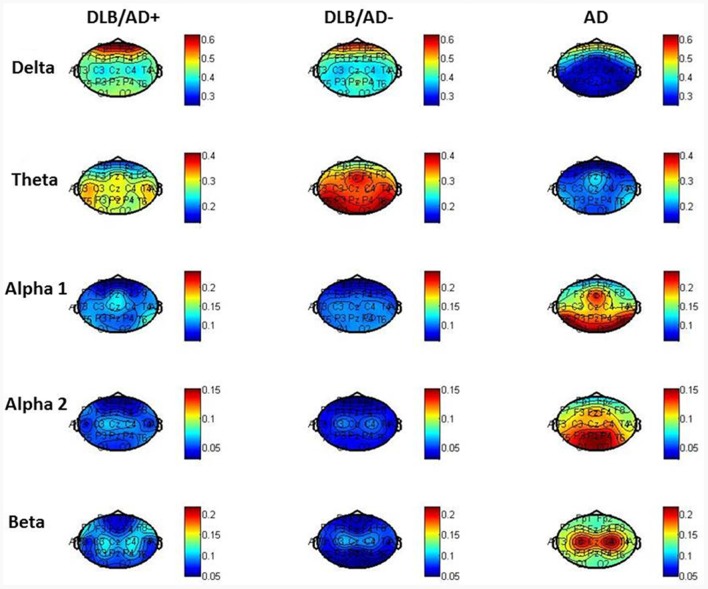
Relative power per frequency band per channel.

The mean relative power of all EEG channels per frequency band is shown in Table 4. Figure 2 shows the distribution of the activity per frequency band across the 21 channels in head plots. Both DLB/AD− and DLB/AD+ show more (posterior) slow-wave activity (higher relative power in delta and theta bands) compared to AD. The relative power in the faster frequency bands (alpha and beta) as well as the peak frequency is lower in the DLB groups than in AD. The abundance of slow-wave activity is also reflected by the theta/alpha-ratio, which is higher in both DLB groups. The variability of the peak frequency was lower (0.38 Hz) in pure DLB compared to in AD (0.49 Hz, *p* = 0.01), the difference between pure DLB and DLB/AD+ (0.48 Hz) was of borderline significance (*p* = 0.06).

**Table 4:** Quantitative EEG characteristics.

	DLB/AD+	DLB/AD−	AD
	(*n* = 41)	(*n* = 41)	(*n* = 41)
Delta power	0.42 (0.23)	0.41 (0.25)	0.32 (0.18)^ab^
Theta power	0.26 (0.13)	0.34 (0.19)	0.18 (0.10)^ab^
Alpha 1 power	0.09 (0.10)	0.07 (0.06)	0.15 (0.15)^ab^
Alpha 2 power	0.04 (0.04)	0.04 (0.03)	0.09 (0.09)^ab^
Beta power	0.08 (0.08)	0.06 (0.04)	0.15 (0.04)^ab^
Peak frequency (Hz)	6.9 (1.17)	6.8 (1.03)	8.4 (1.8)^ab^
Peak frequency variability (Hz)	0.48 (0.25)	0.38 (0.22)	0.4 (0.38)^ b^
Theta/alpha-ratio	0.6 (0.2)	0.8 (0.2)	0.4 (0.3)^ab^
Phase lag index (PLI)*	0.137 (0.035)	0.142 (0.038)	0.177 (0.076)^ab^
Minimum spanning tree (MST)*			
Highest degree	0.175 (0.038)	0.188 (0.056)	0.213 (0.063)^ab^
Leaf number	0.550 (0.050)	0.550 (0.081)	0.575 (0.063)^a^
Diameter	0.400 (0.038)	0.413 (0.050)	0.388 (0.056)
Tree hierarchy	0.384 (0.039)	0.387 (0.050)	0.395 (0.049)

Data are median (IQR) ^\textsl a^p < 0.05 for DLB/AD+ vs. AD, ^\textsl b^p < 0.05 for DLB/AD− vs. AD (Kruskal Wallis test). *Alpha band.

No confounders or effect modifying variables were identified in a regression analysis (using age, sex, symptom duration, MMSE score and “medication use”).

#### Quantitative EEG—Functional Connectivity and Network Characteristics

Table 4 shows functional connectivity strength and network characteristics.

A significant difference was found between both DLB groups and AD for global apha-band PLI (lower PLI for both DLB groups compared to AD) and MST highest degree (lower degree for both DLB groups compared to AD). No significant differences were found between the pure DLB and the DLB/AD+ patients.

### Automated Classification

**Figure 3:**
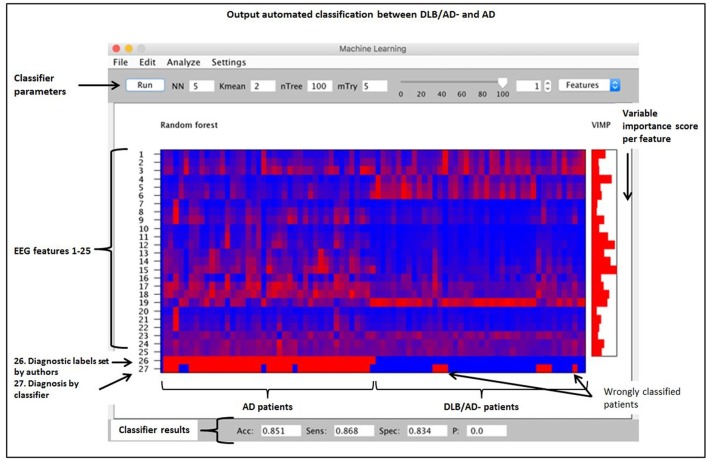
Output of a random forest automated classification between DLB/AD− (pure DLB) and AD. nTree and mTry are parameters used for random forest analysis. Subjects are arranged by diagnosis on the x-axis, no. 1–41 = AD, no. 42–82 = DLB/AD−. Quantitatively (qEEG) features 1 *t/m* 25 as listed in “Materials and Methods” section. VIMP = variable importance score, Acc = accuracy, Sens = sensitivity, Spec = specificity, p = *p*-value.

An automated classification based on only qEEG features between pure DLB and AD reached an accuracy of 85% (sensitivity 87%, specificity 83%). Between DLB/AD+ and AD, the accuracy of the classifier was 74% (sensitivity 70%, specificity 79%). Accuracy for the discrimination between DLB/AD+ and DLB/AD− was 52% (only just above chance performance). The EEG features with the highest VIMP scores were theta/alpha-ratio for DLB/AD+ vs. AD, and beta power for DLB/AD− vs. AD. Figure 3 shows an example of the output of the random forest classification between DLB/AD− and AD, including VIMP scores of the used EEG features.

## Discussion

This study shows that visual and quantitative EEG characteristics of patients with “pure” DLB and DLB with concomitant AD-pathology do not differ, but are both different from AD. Both DLB-groups have compared to AD: more pronounced visual EEG abnormalities (such as FIRDA); a lower peak frequency and more slow-wave activity; a decreased strength of alpha functional connectivity—a difference in network organization (degree).

To our knowledge, this is the first study that takes into account concomitant AD pathology when describing EEG characteristics in DLB. Previous studies have shown that DLB-patients with concomitant AD-pathology have a more aggressive disease course compared to pure DLB (Howlett et al., 2015; Graff-Radford et al., 2016; Lemstra et al., 2017). In line with these findings, our DLB/AD+-group had the lowest MMSE score. While we hypothesized that EEG disturbances would also be more outspoken in these cases, this could not be confirmed by our data. On the contrary, both DLB/AD− and DLB/AD+ have more severe EEG abnormalities than AD patients. A possible explanation for our findings is that DLB pathology “drives” the EEG abnormalities. It has been suggested that in DLB, not cell death, but rather α-synuclein aggregate-related synaptic dysfunction is related to clinical symptoms (Schulz-Schaeffer, 2010). In particular the cholinergic deficit is present earlier and more widespread in DLB compared to AD. Loss of cholinergic activity has been observed in the thalamus and all neocortical regions (Tiraboschi et al., 2002; Francis and Perry, 2007). In a pathological study of patients with Parkinson’s disease dementia (PDD) without concomitant AD pathology, a reduction of hippocampal cholinergic activity together with an increased load of α-synucleinopathy in the basal forebrain and hippocampus has suggested that α-synuclein depositions can induce cholinergic dysfunction (Hall et al., 2014). The outspoken EEG-abnormalities in DLB compared to AD have been linked to this more severe cholinergic deficit and to impaired thalamo- and corticocortical communication (Riekkinen et al., 1991; Kai et al., 2005; van Dellen et al., 2015; Bonanni et al., 2016). This pathological mechanism might not be further aggravated by concomitant AD-pathology. Possibly, the unfavorable clinical course of DLB/AD+ compared to pure DLB, is not caused by changes on a functional level but instead more on a structural level, i.e., increased cortical atrophy, as has been suggested by imaging studies (Nedelska et al., 2015; Graff-Radford et al., 2016). The EEG could miss this additional damage caused by AD co-pathology (“dead neurons tell no tales”).

The pronounced EEG abnormalities we found in DLB are in agreement with the available studies that compare DLB, AD and controls (Bonanni et al., 2016; Cromarty et al., 2016). While recent research tends to focus on the quantitative and automated EEG analysis, visual interpretation of EEG has also shown clear differences between DLB and AD. For example, FIRDA has been described more in DLB compared to in AD (Roks et al., 2008; Lee et al., 2015). This difference is confirmed by our results, where FIRDA is seen significantly more often in both DLB groups (20% and 29%) compared to in AD (2%; Figure 2). For qEEG, the low peak frequency and high relative power in theta and delta bands we found in DLB, are agreement with existing literature. An exception is the peak frequency variability, which has been described to be high in DLB (Bonanni et al., 2008, 2016). This could not be confirmed by our study: the variability (SD of peak frequency over 16 epochs per patient) even seemed higher in both AD and DLB/AD+, compared to pure DLB. These results should be interpreted with some caution since only the pre-selected artifact-free epochs (~40 s of EEG recording) were used to assess variability. Previous studies (Bonanni et al., 2008, 2016) have used larger numbers of epochs. However a publication dedicated to this question states that evaluation of peak frequency variability does not require recordings longer than 20 s (Maltez et al., 2004). Our findings warrant more studies in different cohorts to assess the value of the peak frequency variability in DLB.

High diagnostic accuracies (~90%) have been previously described for automated EEG analyses for the discrimination of DLB from AD and controls (Bonanni et al., 2016; Dauwan et al., 2016a). Our results support the potential of these techniques, although for the clinically difficult discrimination between DLB/AD+ and AD the diagnostic accuracy was a bit lower than for DLB/AD− vs. AD. An advantage of the random forest machine learning technique is that it randomly selects a sample of the data for each decision tree. Therefore, there is no need for a separate dataset for cross-validation (Breiman, 1999) and the automated analysis supports our results for the diagnostic value of (q)EEG.

Our study supports the value of EEG as a true biomarker of Lewy body pathology in dementia. When considering all qEEG data, spectral analysis (such as beta power and theta/alpha ratio) provided more discriminative value than the more advanced analyses of networks and connectivity. Therefore, visual EEG analysis combined with spectral analysis could be the cornerstone of the clinical EEG interpretation, making the diagnostic test low-cost, easy to interpret and widely available. A drawback of visual EEG can be low specificity (Cromarty et al., 2016). However, our study identified (q)EEG features that are valuable for the differentiation between AD and DLB, e.g., FIRDA and the theta/alpha ratio. Future research should focus on identifying cut-off values, e.g., for peak frequency or theta/alpha ratio, that help to classify a patient in one of the diagnostic groups. Possibly the development of a specific EEG-DLB scale could help improve diagnostic value. Validation in a separate dataset (preferably with pathologically confirmed cases) would be feasible to incorporate this in clinical practice.

Currently EEG can have added value, especially in the more complex cases when there is a clinical suspicion of DLB, such as the DLB/AD+ cases, where (CSF) AD biomarkers are not helpful and/or DLB-patients with an initially negative DAT SPECT (van der Zande et al., 2016). An abnormal EEG might be an early marker of DLB (Bonanni et al., 2008). To study EEGs in non-demented patient groups at risk for DLB, for example patients with mild cognitive impairment (MCI) or REM sleep behavior disorder, would be of interest. Furthermore, EEG will have to be related to clinical outcome measures in longitudinal studies, to assess its possibilities as a response marker for treatment.

Strength of this study is the availability of three groups of relatively large size (*n* = 123 in total) from a well-characterized clinical cohort. The groups were well matched for age, sex and disease duration. Especially for the DLB/AD+-cases, the sample is large compared to other studies. It is the first study to link EEG characteristics to CSF biomarkers in DLB. Another strength is the combination of visual, quantitative and automated EEG analyses in one study.

The study had some limitations: first, like in other clinical and imaging studies, pathological confirmation of the diagnosis was lacking. However, diagnoses were made in an expert consensus meeting. When a DAT-SPECT scan was performed in DLB patients, it supported the clinical diagnosis (McKeith et al., 2017). Second, some of the patients used medication that could have influenced the EEG. However, benzodiazepines and antidepressants, the two types of medication that were used most by the DLB/AD+ group, are associated with fast (beta band) activity (Blume, 2006), while in the DLB groups a lower beta power has been found. In a regression analysis medication use was not identified as a confounder or effect-modifying variable. The visual selection of the epochs might have influenced our results, although this has been performed in a standardized manner and overall effects of epoch selection seem to be small (van Diessen et al., 2015). 21-channel EEGs can be used to study connectivity and networks, however the number of channels limits the analysis of specific anatomical regions (van Dellen et al., 2015).

In conclusion, our study shows differences in EEG characteristics between DLB and AD, but not between DLB/AD+ and DLB/AD−. These findings are of importance because: (1) in DLB, concomitant AD pathology does not seem to influence the EEG, which suggests that the Lewy body pathology “drives” the outspoken EEG abnormalities. (2) EEG can be a valuable contribution to differentiate DLB from AD, especially in the DLB/AD+ cases, where the AD-biomarkers are not helpful.

## Author Contributions

JZ: study conception and design, data acquistion, data analysis and interpretation, drafting the article. AG, PS, CJS and AL: study conception and design, critical revision of the article. IS: data analysis and interpretation, critical revision of the article.

## Conflict of Interest Statement

The authors declare that the research was conducted in the absence of any commercial or financial relationships that could be construed as a potential conflict of interest. The reviewer YJ and handling Editor declared their shared affiliation.

## Footnotes

^1^http://home.kpn.nl/stam7883/brainwave.html

## References

[B1] Blume W. T. (2006). Drug effects on EEG. J. Clin. Neurophysiol. 23, 306–311. 10.1097/01.wnp.0000229137.94384.fa 16885705

[B2] Bonanni L. Franciotti R. Nobili F. Kramberger M. G. Taylor J. P. Garcia-Ptacek S.. (2016). EEG markers of dementia with lewy bodies: a multicenter cohort study. J. Alzheimers Dis. 54, 1649–1657. 10.3233/JAD-160435 27589528

[B3] Bonanni L. Thomas A. Tiraboschi P. Perfetti B. Varanese S. Onofrj M. (2008). EEG comparisons in early Alzheimer’s disease, dementia with Lewy bodies and Parkinson’s disease with dementia patients with a 2-year follow-up. Brain 131, 690–705. 10.1093/brain/awm322 18202105

[B4] Breiman L. (1999). Random forest. Mach. Learn. 45, 5–35. 10.1023/A:1010933404324

[B5] Cockrell J. R. Folstein M. F. (1988). Mini-mental state examination (MMSE). Psychopharmacol. Bull. 24, 689–692. 3249771

[B6] Cromarty R. A. Elder G. J. Graziadio S. Baker M. Bonanni L. Onofrj M.. (2016). Neurophysiological biomarkers for Lewy body dementias. Clin. Neurophysiol. 127, 349–359. 10.1016/j.clinph.2015.06.020 26183755 PMC4727506

[B7] Cummings J. L. (1997). The neuropsychiatric inventory: assessing psychopathology in dementia patients. Neurology 48, S10–S16. 10.1212/wnl.48.5_suppl_6.10s 9153155

[B8] Dauwan M. van Dellen E. van Boxtel L. van Straaten E. C. de Waal H. Lemstra A. W.. (2016a). EEG-directed connectivity from posterior brain regions is decreased in dementia with Lewy bodies: a comparison with Alzheimer’s disease and controls. Neurobiol. Aging 41, 122–129. 10.1016/j.neurobiolaging.2016.02.017 27103525

[B9] Dauwan M. van der Zande J. J. van Dellen E. Sommer I. E. Scheltens P. Lemstra A. W.. (2016b). Random forest to differentiate dementia with Lewy bodies from Alzheimer’s disease. Alzheimers Dement. 4, 99–106. 10.1016/j.dadm.2016.07.003 27722196 PMC5050257

[B10] Duits F. H. Teunissen C. E. Bouwman F. H. Visser P. J. Mattsson N. Zetterberg H.. (2014). The cerebrospinal fluid “Alzheimer profile”: easily said, but what does it mean? Alzheimers Dement. 10, 713.e2–723.e2. 10.1016/j.jalz.2013.12.023 24721526

[B11] Francis P. T. Perry E. K. (2007). Cholinergic and other neurotransmitter mechanisms in Parkinson’s disease, Parkinson’s disease dementia, and dementia with Lewy bodies. Mov. Disord. 22, S351–S357. 10.1002/mds.21683 18175396

[B12] Gasser T. Bächer P. Steinberg H. (1985). Test-retest reliability of spectral parameters of the EEG. Electroencephalogr. Clin. Neurophysiol. 60, 312–319. 10.1016/0013-4694(85)90005-7 2579798

[B13] Geurts P. Irrthum A. Wehenkel L. (2009). Supervised learning with decision tree-based methods in computational and systems biology. Mol. Biosyst. 5, 1593–1605. 10.1039/b907946g 20023720

[B14] Graff-Radford J. Lesnick T. G. Boeve B. F. Przybelski S. A. Jones D. T. Senjem M. L.. (2016). Predicting survival in dementia with lewy bodies with hippocampal volumetry. Mov. Disord. 31, 989–994. 10.1002/mds.26666 27214825 PMC4931944

[B15] Hagemann D. Naumann E. (2001). The effects of ocular artifacts on (lateralized) broadband power in the EEG. Clin. Neurophysiol. 112, 215–231. 10.1016/s1388-2457(00)00541-1 11165523

[B16] Hall H. Reyes S. Landeck N. Bye C. Leanza G. Double K.. (2014). Hippocampal Lewy pathology and cholinergic dysfunction are associated with dementia in Parkinson’s disease. Brain 137, 2493–2508. 10.1093/brain/awu193 25062696

[B17] Hjorth B. (1975). An on-line transformation of EEG scalp potentials into orthogonal source derivations. Electroencephalogr. Clin. Neurophysiol. 39, 526–530. 10.1016/0013-4694(75)90056-5 52448

[B18] Howlett D. R. Whitfield D. Johnson M. Attems J. O’Brien J. T. Aarsland D.. (2015). Regional multiple pathology scores are associated with cognitive decline in lewy body dementias. Brain Pathol. 25, 401–408. 10.1111/bpa.12182 25103200 PMC8029273

[B19] Jellinger K. A. (2004). Influence of Alzheimer pathology on clinical diagnostic accuracy in dementia with Lewy bodies. Neurology 62:160; author reply 160. 10.1212/wnl.62.1.160 14718731

[B20] Jellinger K. A. Attems J. (2008). Prevalence and impact of vascular and Alzheimer pathologies in Lewy body disease. Acta Neuropathol. 115, 427–436. 10.1007/s00401-008-0347-5 18273624

[B21] Kai T. Asai Y. Sakuma K. Koeda T. Nakashima K. (2005). Quantitative electroencephalogram analysis in dementia with Lewy bodies and Alzheimer’s disease. J. Neurol. Sci. 237, 89–95. 10.1016/j.jns.2005.05.017 16019033

[B22] Lee H. Brekelmans G. J. Roks G. (2015). The EEG as a diagnostic tool in distinguishing between dementia with Lewy bodies and Alzheimer’s disease. Clin. Neurophysiol. 126, 1735–1739. 10.1016/j.clinph.2014.11.021 25534493

[B23] Lemstra A. W. de Beer M. H. Teunissen C. E. Schreuder C. Scheltens P. van der Flier W. M.. (2017). Concomitant AD pathology affects clinical manifestation and survival in dementia with Lewy bodies. J. Neurol. Neurosurg. Psychiatry 88, 113–118. 10.1136/jnnp-2016-313775 27794030

[B24] Liedorp M. van der Flier W. M. Hoogervorst E. L. Scheltens P. Stam C. J. (2009). Associations between patterns of EEG abnormalities and diagnosis in a large memory clinic cohort. Dement. Geriatr Cogn. Disord. 27, 18–23. 10.1159/000182422 19088474

[B25] Maltez J. Hyllienmark L. Nikulin V. V. Brismar T. (2004). Time course and variability of power in different frequency bands of EEG during resting conditions. Neurophysiol. Clin. 34, 195–202. 10.1016/j.neucli.2004.09.003 15639128

[B26] McCleery J. Morgan S. Bradley K. M. Noel-Storr A. H. Ansorge O. Hyde C. (2015). Dopamine transporter imaging for the diagnosis of dementia with Lewy bodies. Cochrane Database Syst. Rev. 1:CD010633. 10.1002/14651858.CD010633.pub2 25632881 PMC7079709

[B27] McKeith I. G. Boeve B. F. Dickson D. W. Halliday G. Taylor J. P. Weintraub D.. (2017). Diagnosis and management of dementia with Lewy bodies: fourth consensus report of the DLB Consortium. Neurology 89, 88–100. 10.1212/WNL.0000000000004058 28592453 PMC5496518

[B28] McKeith I. G. Dickson D. W. Lowe J. Emre M. O’Brien J. T. Feldman H.. (2005). Diagnosis and management of dementia with Lewy bodies: third report of the DLB Consortium. Neurology 65, 1863–1872. 10.1212/01.wnl.0000187889.17253.b1 16237129

[B29] McKhann G. M. Knopman D. S. Chertkow H. Hyman B. T. Jack C. R. Jr. Kawas C. H.. (2011). The diagnosis of dementia due to Alzheimer’s disease: recommendations from the national institute on aging-Alzheimer’s association workgroups on diagnostic guidelines for Alzheimer’s disease. Alzheimers Dement. 7, 263–269. 10.1016/j.jalz.2011.03.005 21514250 PMC3312024

[B30] Nedelska Z. Ferman T. J. Boeve B. F. Przybelski S. A. Lesnick T. G. Murray M. E.. (2015). Pattern of brain atrophy rates in autopsy-confirmed dementia with Lewy bodies. Neurobiol. Aging 36, 452–461. 10.1016/j.neurobiolaging.2014.07.005 25128280 PMC4268128

[B31] Riekkinen P. Buzsaki G. Riekkinen P. Jr. Soininen H. Partanen J. (1991). The cholinergic system and EEG slow waves. Electroencephalogr. Clin. Neurophysiol. 78, 89–96. 10.1016/0013-4694(91)90107-f 1704840

[B32] Roks G. Korf E. S. van der Flier W. M. Scheltens P. Stam C. J. (2008). The use of EEG in the diagnosis of dementia with Lewy bodies. J. Neurol. Neurosurg. Psychiatry 79, 377–380. 10.1136/jnnp.2007.125385 17682010

[B33] Scheltens P. Blennow K. Breteler M. M. de Strooper B. Frisoni G. B. Salloway S.. (2016). Alzheimer’s disease. Lancet 388, 505–517. 10.1016/S0140-6736(15)01124-1 26921134

[B34] Schulz-Schaeffer W. J. (2010). The synaptic pathology of alpha-synuclein aggregation in dementia with Lewy bodies, Parkinson’s disease and Parkinson’s disease dementia. Acta Neuropathol. 120, 131–143. 10.1007/s00401-010-0711-0 20563819 PMC2892607

[B35] Stam C. J. Nolte G. Daffertshofer A. (2007). Phase lag index: assessment of functional connectivity from multi channel EEG and MEG with diminished bias from common sources. Hum. Brain Mapp. 28, 1178–1193. 10.1002/hbm.20346 17266107 PMC6871367

[B36] Tiraboschi P. Hansen L. A. Alford M. Merdes A. Masliah E. Thal L. J.. (2002). Early and widespread cholinergic losses differentiate dementia with Lewy bodies from Alzheimer disease. Arch. Gen. Psychiatry 59, 946–951. 10.1001/archpsyc.59.10.946 12365882

[B37] Toledo J. B. Cairns N. J. Da X. Chen K. Carter D. Fleisher A.. (2013). Clinical and multimodal biomarker correlates of ADNI neuropathological findings. Acta Neuropathol. Commun. 1:65. 10.1186/2051-5960-1-65 24252435 PMC3893373

[B38] Treglia G. Cason E. (2012). Diagnostic performance of myocardial innervation imaging using MIBG scintigraphy in differential diagnosis between dementia with lewy bodies and other dementias: a systematic review and a meta-analysis. J. Neuroimaging 22, 111–117. 10.1111/j.1552-6569.2010.00532.x 21091814

[B39] van Dellen E. de Waal H. van der Flier W. M. Lemstra A. W. Slooter A. J. Smits L. L.. (2015). Loss of EEG network efficiency is related to cognitive impairment in dementia with lewy bodies. Mov. Disord. 30, 1785–1793. 10.1002/mds.26309 26179663

[B40] van der Flier W. M. Pijnenburg Y. A. Prins N. Lemstra A. W. Bouwman F. H. Teunissen C. E.. (2014). Optimizing patient care and research: the Amsterdam Dementia Cohort. J. Alzheimers Dis. 41, 313–327. 10.3233/JAD-132306 24614907

[B41] van der Zande J. J. Booij J. Scheltens P. Raijmakers P. G. Lemstra A. W. (2016). [^123^]FP-CIT SPECT scans initially rated as normal became abnormal over time in patients with probable dementia with Lewy bodies. Eur. J. Nucl. Med. Mol. Imaging 43, 1060–1066. 10.1007/s00259-016-3312-x 26830298 PMC4844648

[B42] van Diessen E. Numan T. van Dellen E. van der Kooi A. W. Boersma M. Hofman D.. (2015). Opportunities and methodological challenges in EEG and MEG resting state functional brain network research. Clin. Neurophysiol. 126, 1468–1481. 10.1016/j.clinph.2014.11.018 25511636

[B43] Whitham E. M. Pope K. J. Fitzgibbon S. P. Lewis T. Clark C. R. Loveless S.. (2007). Scalp electrical recording during paralysis: quantitative evidence that EEG frequencies above 20 Hz are contaminated by EMG. Clin. Neurophysiol. 118, 1877–1888. 10.1016/j.clinph.2007.04.027 17574912

[B44] Zaccai J. McCracken C. Brayne C. (2005). A systematic review of prevalence and incidence studies of dementia with Lewy bodies. Age Ageing 34, 561–566. 10.1093/ageing/afi190 16267179

